# Cancer in a drop: Liquid biopsy highlights from the San Antonio Breast Cancer Symposium (SABCS) 2025

**DOI:** 10.1016/j.jlb.2026.100463

**Published:** 2026-03-27

**Authors:** Eleonora Nicolò, Konstantinos Venetis, Enes Erul, Pasquale Pisapia, Letizia Pontolillo, Carolina Reduzzi

**Affiliations:** a Department of Medicine, Division of Hematology-Oncology, Weill Cornell Medicine, New York, NY, USA; b Division of Pathology, European Institute of Oncology IRCCS, Milan, Italy; c Department of Medical Oncology, Ankara University Faculty of Medicine, Ankara University, Ankara, Türkiye; d Ankara University Cancer Institute, Ankara University, Ankara, Türkiye; e Department of Public Health, University of Naples Federico II, Naples, Italy; f Department of Translational Medicine and Surgery, Università Cattolica del Sacro Cuore, Rome, Italy

**Keywords:** Breast cancer, Liquid biopsy, Circulating tumor DNA, Minimal residual disease, Resistance mechanisms

## 1 Introduction

The San Antonio Breast Cancer Symposium (SABCS) 2025 reaffirmed liquid biopsy as a transformative tool in breast cancer care. From early detection to monitoring minimal residual disease (MRD), predicting response, and decoding resistance, circulating biomarkers are reshaping clinical paradigms. This editorial synthesizes key presentations from the SABCS 2025, highlighting how blood-based assays are moving from research to real-world impact. To provide a focused overview, we included all abstracts on liquid biopsy in breast cancer that were presented orally, as these represent the most clinically relevant contributions selected by the symposium. Abstracts presented exclusively as posters were not included.

## 2 Early breast cancer detection: proteomics meets Artificial Intelligence (AI)

Early detection remains the cornerstone of improving breast cancer outcomes. At SABCS 2025, a novel deep proteomics–AI platform demonstrated strong potential for early detection using <1 mL of plasma. Leveraging label-free quantitative mass spectrometry and a proprietary breast cancer–specific spectral library, the platform profiled up to 7500 proteins per patient and identified differentially expressed proteins distinguishing early-stage breast cancer (EBC) from healthy controls. Trained on 845 individuals and validated in an independent cohort of 397, the classifier achieved >92% sensitivity and specificity across all stages, molecular, and pathological subtypes [1]. Pending further validation, this high-depth, low-volume plasma assay could improve screening strategies and accelerate clinical implementation of liquid biopsy for EBC detection.

## 3 Early breast cancer: response prediction and MRD monitoring

Recent advances in neoadjuvant chemo-immunotherapy have improved outcomes for patients with early-stage triple-negative breast cancer (TNBC), yet many patients do not achieve pathological complete response (pCR) or remain at high risk of recurrence, underscoring the need for robust, minimally invasive biomarkers to guide treatment decisions. Circulating immune and tumor-derived biomarkers have emerged as powerful predictors of response and long-term outcomes. Multi-omic immune profiling reveals that response to immune checkpoint blockade is strongly influenced by the baseline systemic immune state [2,3]. Patients achieving pCR consistently exhibit immune-enriched profiles characterized by activated adaptive immunity, including higher levels of activated memory CD4^+^ and CD8^+^ T cells, B cells, CD39^+^ CD8^+^ T cells, and lymphocyte-driven interferon signaling, alongside reduced immunosuppressive myeloid and exhausted T-cell populations. In contrast, non-responders demonstrate pre-existing anti-immune states marked by suppressive neutrophils, exhausted T cells, ineffective interferon responses, and pro-tumorigenic circulating proteins such as CCL23 and ANXA4. Dynamic immune evolution during therapy further distinguishes responders, who transition toward primed memory T-cell states and mount effective TNFα/NFκB-driven inflammatory responses, while non-responders fail to sustain productive immune activation.

Complementing immune profiling, circulating tumor DNA (ctDNA) analysis provides orthogonal and highly prognostic information. Tumor-informed and tumor-agnostic ctDNA assays consistently show that baseline ctDNA is detectable in most TNBC patients [4,5], with rapid clearance during neoadjuvant therapy strongly associated with pCR and excellent event-free survival [4]. Conversely, persistent or newly detectable ctDNA during or after therapy predicts poor outcomes (up to 30-fold higher risk of recurrence), often preceding clinical relapse by several months [5,6]. Notably, ctDNA positivity after surgery confers an exceptionally high risk of distant recurrence, regardless of pCR status, highlighting limitations of surgical endpoints alone [5]. Tissue-free methylation-based assays show high concordance (95.4%) with tissue-informed approach based on digital droplet PCR while offering earlier detection and simpler workflows [6]. Collectively, these findings indicate that both immune responsiveness and therapeutic resistance in TNBC are largely pre-determined by baseline biology but dynamically measurable through peripheral blood. Integration of circulating immune signatures with longitudinal ctDNA monitoring offers a powerful framework for patient stratification, real-time treatment adaptation, and rational escalation or de-escalation of neoadjuvant chemo-immunotherapy to improve cure rates while minimizing unnecessary toxicity.

Beyond TNBC, ctDNA has become a powerful lens on response and MRD across subtypes. In HER2-positive EBC, the PHERGain translational analysis embedded a tissue-free, methylation-based assay within a PET-guided de-escalation neoadjuvant trial. Overall, ctDNA was detected at baseline in 71% of patients, with higher detection rates among those with stage III disease and/or nodal involvement. Among those ctDNA-positive at baseline, 76% cleared ctDNA by cycle 3 and 83% by pre-surgery, leaving only ∼12% with persistent ctDNA. Baseline ctDNA status showed no association with pCR, whereas ctDNA clearance strongly correlated with pCR; notably, no patient with presurgical ctDNA positivity achieved pCR. Regarding outcomes, ctDNA detection at any time point as well as lack of ctDNA clearance during neoadjuvant therapy were associated with worse 3-year invasive disease-free survival (iDFS), highlighting dynamic ctDNA monitoring as a robust, orthogonal marker of residual risk [7]. In the HR-positive/HER2-negative setting, a PALLAS sub-analysis applied a tumor-informed assay to >1000 plasma samples from patients receiving adjuvant endocrine therapy with or without palbociclib. Across timepoints, ctDNA detection was relatively infrequent (<10%) but highly informative: patients with at least one MRD-positive sample had substantially higher recurrence rates, whereas sustained MRD negativity identified a group with very low event rates, positioning ctDNA as a robust prognostic tool in this population [8]. Interesting preliminary results were reported from the ctDNA analysis, using the tumor-informed RaDaR assay, in the CLEVER trial [9]. CLEVER is a phase II study including patients with high-risk EBC with detectable disseminated tumor cells (DTCs) via bone marrow after completion of definitive treatment (except endocrine therapy), investigating targeting DTCs to prevent recurrence [10]. Among 51 patients included, 32 were evaluable for ctDNA and two were ctDNA-positive. These were the only two patients that experienced recurrence in the trial, and both did not clear the DTCs, suggesting that ctDNA detection might be related to DTC reactivation prior to recurrence.

The prospective phase II LEADER trial moves MRD from observation to intervention, testing ribociclib for one year in patients with HR-positive/HER2-negative BC who were ctDNA-positive during adjuvant endocrine therapy. Using a tumor-informed assay, ctDNA positivity occurred in 11% of patients (15/140), and 10 received ribociclib. In this cohort, ctDNA clearance on ribociclib correlated with longer distant recurrence-free survival (DRFS), although late recurrences still occurred, underscoring the resilience of micrometastatic ER-positive disease. The remaining 89% of patients were MRD-negative, with remarkably high negative predictive values: recurrence-free survival, 100% and 99% at 6 and 12 months; DRFS, 100% at both time points [11].

Finally, the I-SURV study reminds us of the importance of MRD surveillance as a lived experience for patients, beyond its biological dimension. In this prospective survey study of patients undergoing ctDNA testing in the adjuvant setting, patient-reported quality of life measures remained similar before and after receiving test results. Twenty-one patients completed paired surveys around their first ctDNA result; only one was MRD-positive. Among the 20 MRD-negative patients, five moved from high to low fear-of-recurrence categories and three entered the top quartile for well-being, and >80% overall valued ongoing ctDNA testing [12]. Collectively, these studies show how ctDNA-defined MRD can inform de-escalation, risk stratification, interventional strategies, and the patient experience of survivorship.

Beyond ctDNA, one study investigated CTCs during adjuvant radiotherapy (RT) in EBC showing that, while 24% (61/249) of patients were CTC-positive before RT, 89% cleared CTCs by the end of RT. In addition, in CTC-positive patients, an increase in HER2 expression on CTCs was observed, both in patients with HER2-positive and HER2-negative BC. These results suggest that CTCs might be a useful tool to monitor response to RT and to assess phenotypic evolution of the tumor under treatment pressure [13].

## 4 Metastatic breast cancer: decoding response and resistance through dynamic biomarkers

Multiple studies presented at SABCS 2025 leveraged ctDNA to dissect mechanisms of response and resistance in HR-positive/HER2-negative metastatic breast cancer (MBC), highlighting its role in assessing early treatment efficacy and resistance evolution. In the phase III EMBER-3 trial, early ctDNA dynamics assessed by the Guardant360 assay from baseline to 4 weeks emerged as a robust pharmacodynamic marker. Among patients with *ESR1*-mutant disease, imlunestrant induced a greater median reduction in overall ctDNA burden compared with standard endocrine therapy (−74% vs −40%), with near-complete suppression of *ESR1*-mutant alleles (−98% vs −75%). Across all patients, the addition of abemaciclib further deepened ctDNA decline (−58% vs −37%), irrespective of *ESR1* mutation status. A ≥50% ctDNA reduction at four weeks was associated with longer progression-free survival (PFS), although assigned treatment remained the primary determinant of outcome [14]. The phase III SERENA-6 trial demonstrated how serial ctDNA monitoring can directly inform treatment decisions. Early detection of emergent *ESR1* mutations during first-line aromatase inhibitor (AI) plus CDK4/6 inhibition enabled an early switch to camizestrant, resulting in rapid molecular suppression of *ESR1*-mutant ctDNA (median −100% at 8 weeks vs +66.7% with continued AI; p < 0.00001) [15]. Targeting PI3K/AKT signaling was further refined in the CAPItello-291 trial. In an exploratory ctDNA analysis, concordance between tumor tissue and plasma was 83.3%, with actionable *PIK3CA/AKT1/PTEN* alterations detected by ctDNA in 40% of patients, including 10% identified exclusively in plasma. Methylation-based tumor fraction (TF) was significantly lower in tissue-only cases (p < 0.0001), consistent with low tumor shedding. Despite assay discordance, most notable for *PTEN*, the clinical benefit of capivasertib plus fulvestrant in ctDNA-altered patients was consistent with tissue-based results and independent of *ESR1* mutation status [16]. Mechanisms of acquired resistance were explored in the phase 1b CAPItello-292 study using paired baseline and end-of-treatment ctDNA. More than half of patients acquired new genomic alterations at progression, predominantly involving activation of alternative pathways such as cell cycle regulation, RTK, RAS–MAPK, and mTOR signaling, while few involved PIK3CA/PTEN alterations retaining sensitivity to capivasertib. Functional CRISPR screens confirmed loss of TSC1, TSC2, and STK11 as key resistance drivers [17]. Finally, long-term analyses from MONALEESA studies [18] and biomarker data from INAVO120 [19] highlighted the prognostic value of baseline ctDNA. Long-term first-line responders to ribociclib plus endocrine therapy showed lower ctDNA fractions and fewer adverse genomic features, including *TP53* and *CCND1* alterations. In INAVO120, benefit from adding inavolisib was independent of *ESR1* mutation status and *PTEN* alterations, and consistent across ctDNA tumor fraction subgroups.

In HER2-positive MBC, the MOONRISE study introduced the concept of MRD monitoring in the metastatic setting to identify exceptional responders to first-line targeted therapy (PFS ≥3 years). Using MAESTRO, an ultra-sensitive tumor-informed assay, 147 samples from 63 patients were analyzed (40 “exceptional responders”, 23 “conventional responders”). At the 1-year landmark, none of the exceptional responders were MRD-positive, while 75% of conventional responders were (p < 0.001). Persistent MRD negativity or early clearance correlated with outstanding long-term outcomes, whereas MRD positivity predicted progression, often years in advance. Notably, 30% of MRD-positive samples had TF < 100 ppm, below the detection limits of first-generation MRD assays, highlighting misclassification risk [20].

Together, these data position ctDNA as a central tool to quantify response, anticipate resistance, guide therapeutic sequencing, and potentially inform therapy de-escalation in future trials.

## 5 Disease characterization and tumor evolution

Two studies used liquid biopsy assays to characterize the genomic landscape of invasive lobular carcinoma (ILC), leveraging large real-world data sets. In the first study, whole-exome sequencing was performed as part of the Signatera workflow on the primary tumor of 1370 patients with HR-positive/HER2-negative ILC. This analysis confirmed the central role of *CDH1* loss in ILC and *TP53* and *ERBB2* mutations as being associated with high-grade disease and shorter DRFS. The study also explored mutational signatures and identified five subgroups, underscoring the molecular heterogeneity of ILC and providing new insights into ILC biology [21]. The second study, which included both patients with metastatic ILC (n = 178) and non-special type (NST, n = 1037) breast carcinomas, performed a comprehensive genomic profiling through Guardant360 testing. The majority of patients (93.5%) had detectable alterations, with no difference among subtypes. In addition to *CDH1*, alterations in *PIK3CA*, *ERBB2*, and *RB1* were more common in ILC than NST, and in 66.3% of ILC patients, clinically relevant mutations were detected, supporting the use of liquid biopsy alteration detection in ILC [22].

Tumor evolution was another focus, with presentations showing how advanced liquid biopsy approaches uncover the dynamic evolutionary trajectories of MBC. The first study employed a capture-based enzymatic ctDNA methylation assay to develop three machine-learning classifiers distinguishing ER + vs ER–, HER2+ vs HER2–, and TNBC vs non-TNBC. In 191 plasma samples from 86 patients, the assay identified tissue-defined subtype switching with 89% accuracy, including transitions from ER+/HER2– to TNBC. Importantly, it detected mixed phenotypes in 8% of cases, indicating coexistence of divergent subclones and highlighting liquid biopsy's advantage over single-site biopsies for tracking tumor phenotype during therapy [23]. A longitudinal study of HR-positive/HER2–negative MBC used serial ctDNA profiling from the EMBRACE cohort to characterize progression from early metastatic disease to lethal end-stage cancer. Among 52 patients with paired samples there was an enrichment in genomic alterations from early to lethal MBC. Among patients with TF >1% at both time points, lethal MBC showed dramatic enrichment of resistance-associated alterations, including *RB1* (12%→46%), *CDKN2A* (4%→31%), *ESR1* (27%→65%), *ERBB2* (15%→42%), and *NF1* (12%→50%). Nearly two-thirds of acquired events were copy-number alterations, revealing profound genomic instability [24]. Together, these studies underscore the value of serial ctDNA monitoring to capture phenotypic shifts and convergent resistance evolution in advanced breast cancer.

## 6 Conclusions

**Fig. 1:**
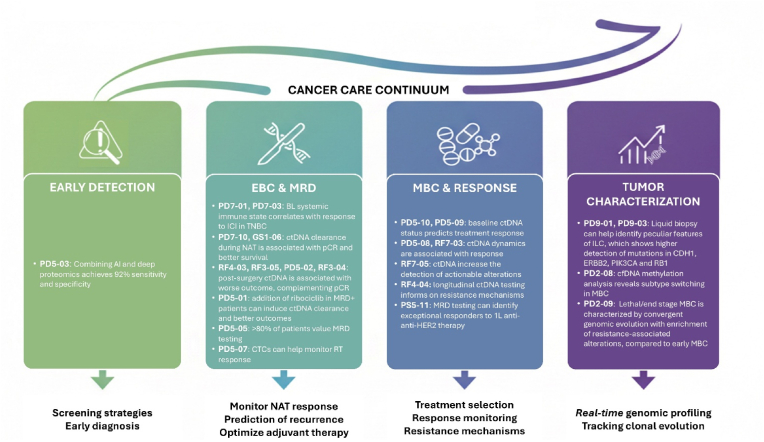
The liquid biopsy roadmap in breast cancer care: key highlights from SABCS 2025. The figure summarizes key studies focused on liquid biopsy presented at SABCS 2025 and their applications across the cancer care continuum—from early detection and MRD monitoring to treatment selection, response assessment, and tumor characterization—along with clinical implications for early diagnosis, risk stratification, therapy optimization, and resistance monitoring. Abbreviations: AI, Artificial Intelligence; EBC, Early Breast Cancer; MRD, Minimal Residual Disease; BL, baseline, ICIs, immune checkpoint inhibitors, NAT, Neoadjuvant Therapy; TNBC, Triple-Negative Breast Cancer; pCR, Pathologic Complete Response; ctDNA, Circulating Tumor DNA; CTCs, Circulating Tumor Cells; RT, Radiotherapy; iDFS, Invasive Disease-Free Survival; HR, Hormone Receptor; HER2, Human Epidermal Growth Factor Receptor 2; MBC, Metastatic Breast Cancer; DRFS, Distant Recurrence-Free Survival; ILC, Invasive Lobular Carcinoma.

The data presented at SABCS 2025 clearly demonstrate that liquid biopsy is reshaping our approach to breast cancer across the entire disease continuum (Fig. 1). Blood-based assays, including ctDNA, CTCs, immune profiling, and emerging proteomic platforms, are transitioning into clinical practice, offering unprecedented opportunities for precision medicine. These tools enable refined risk stratification, guide therapeutic decisions, deepen our understanding of disease biology and evolution, and open opportunities for early intervention while improving survivorship care. As technologies mature and prospective trials expand, the clinical utility of liquid biopsy is becoming increasingly evident, solidifying its role as a central element of precision oncology in breast cancer care.

It is important to note that these insights are based on SABCS 2025 conference abstracts, which have not yet undergone full peer review, and should therefore be interpreted with caution in the context of the currently available evidence. In the short term, several applications are approaching or entering clinical practice. The identification of ctDNA-detected actionable genomic alterations to guide treatment decisions is now routinely used, and the expanded integration of longitudinal monitoring is providing deeper insight into resistance mechanisms that may inform therapeutic strategies. Detection of MRD is also nearing clinical implementation, pending demonstration of its clinical utility, with ongoing efforts evaluating MRD-guided therapeutic approaches. Moreover, robust validation of ctDNA clearance as a surrogate endpoint for treatment response remains necessary. Conversely, proteomic profiling, complex multi-omic signatures, and early detection strategies remain investigational and will require further exploration and validation before influencing routine practice.

## Ethical approval

No ethical approvals or patient consent were necessary for the study.

## Declaration of competing interest

The authors declare the following financial interests/personal relationships which may be considered as potential competing interests: Eleonora Nicolò reports a relationship with Guardant Health Inc that includes: speaking and lecture fees. Konstantinos Venetis reports a relationship with Merck Sharp & Dohme that includes: speaking and lecture fees. Konstantinos Venetis reports a relationship with Roche that includes: speaking and lecture fees. Konstantinos Venetis reports a relationship with AstraZeneca that includes: speaking and lecture fees. Konstantinos Venetis reports a relationship with Veracyte that includes: speaking and lecture fees. Konstantinos Venetis reports a relationship with Johnson & Johnson that includes: speaking and lecture fees. Letizia Pontolillo reports a relationship with Pfizer that includes: travel reimbursement. Letizia Pontolillo reports a relationship with Eli Lilly and Company that includes: travel reimbursement. Letizia Pontolillo reports a relationship with Gilead Sciences Inc that includes: travel reimbursement. Letizia Pontolillo reports a relationship with Menarini Silicon Biosystems that includes: travel reimbursement. Letizia Pontolillo reports a relationship with Daiichi Sankyo Inc that includes: speaking and lecture fees. Letizia Pontolillo reports a relationship with Novartis that includes: speaking and lecture fees. Carolina Reduzzi reports a relationship with Menarini Silicon Biosystems that includes: funding grants. Carolina Reduzzi reports a relationship with ANGLE plc that includes: funding grants. Carolina Reduzzi reports a relationship with Tethis that includes: funding grants. Carolina Reduzzi reports a relationship with Qiagen that includes: funding grants. Eleonora Nicolò: Young Committee Member of the International Society of Liquid Biopsy (ISLB). Uncompensated member of Precision Medicine Action for Cancer (PMAC) Board of Directors. Kostantinos Venetis: Young Committee Member of the International Society of Liquid Biopsy (ISLB). Enes Erul: Young Committee Member of the International Society of Liquid Biopsy (ISLB). Pasquale Pisapia: Young Committee Member of the International Society of Liquid Biopsy (ISLB). Letizia Pontolillo: Young Committee Member of the International Society of Liquid Biopsy (ISLB). Carolina Reduzzi: Young Committee Chair of the International Society of Liquid Biopsy (ISLB). Uncompensated member of Precision Medicine Action for Cancer (PMAC) Board of Directors. If there are other authors, they declare that they have no known competing financial interests or personal relationships that could have appeared to influence the work reported in this paper.
